# Supplementary material for: Peculiar pigment pattern and population profile of a poisonous pufferfish
Source: PeerJ. 2026 Jun 5;14:e21378. doi: 10.7717/peerj.21378 (PMC13245424; doi:10.7717/peerj.21378)
Supplement: Supplemental Information 1 [file peerj-14-21378-s001.pdf]

Table S1: Metadata of *Takifugu exascurus* (mushifugu) and *T. flavipterus* (komonfugu) samples used in this study.

Details of individual samples analyzed in this study. Columns include sample ID, species, population (SJ: Sea of Japan; PO: Pacific Ocean), collection locality, collection date, availability of body pattern image (1: available, 0: not available), pattern metrics (lightness and complexity), availability of mitochondrial D-loop sequence (1: available, 0: not available), whole-genome resequencing data (NGS; 1: available, 0: not available), and BioSample accession numbers (if assigned).

ID	Species	Population	Location	Date	Image	Lightness	Complexity	D-loop	NGS	BioSample
Mj_002	Mushifugu	SJ	Sado Island, Niigata Pref.	2006-06	1	0.38396	0.76110	1	0	
Mj_003	Mushifugu	SJ	Sado Island, Niigata Pref.	2006-06	1	0.37006	0.72597	1	1	SAMD01606002
Mj_004	Mushifugu	SJ	Sado Island, Niigata Pref.	2006-06	1	0.37220	0.74210	1	0	
Mj_005	Mushifugu	SJ	Sado Island, Niigata Pref.	2006-06	1	0.32359	0.73005	1	0	
Mj_006	Mushifugu	SJ	Sado Island, Niigata Pref.	2006-06	1	0.36564	0.77450	1	1	SAMD01606003
Mj_017	Mushifugu	SJ	Hamasaka, Hyogo Pref.	2006-04-27	1	0.39043	0.87624	1	0	
Mj_058	Mushifugu	SJ	Gotsu, Shimane Pref.	2007-06-15	1	0.36045	0.73408	1	0	
Mj_065	Mushifugu	SJ	Kasumi, Hyogo Pref.	2012-06-27	1	0.39179	0.74039	1	1	SAMD01606004
Mj_074	Mushifugu	SJ	Hamasaka, Hyogo Pref.	NA	0	NA	NA	1	0	
Mj_075	Mushifugu	SJ	Hamasaka, Hyogo Pref.	NA	0	NA	NA	1	0	
Mj_076	Mushifugu	SJ	Sado Island, Niigata Pref.	NA	0	NA	NA	1	0	
Mj_091	Mushifugu	SJ	Sado Island, Niigata Pref.	2013-06-05	1	0.38844	0.86359	1	0	
Mj_092	Mushifugu	SJ	Sado Island, Niigata Pref.	2013-06-05	1	0.38061	0.75647	1	0	
Mj_093	Mushifugu	SJ	Sado Island, Niigata Pref.	2013-06-05	1	0.37332	0.82750	1	0	
Mj_098	Mushifugu	SJ	Sado Island, Niigata Pref.	2013-06-05	1	0.33212	0.63052	1	1	SAMD01606005
Mj_099	Mushifugu	SJ	Sado Island, Niigata Pref.	2013-06-05	1	0.30407	0.69828	1	0	
Mj_100	Mushifugu	SJ	Sado Island, Niigata Pref.	2013-06-05	1	0.36573	0.80173	1	0	
Mj_101	Mushifugu	SJ	Sado Island, Niigata Pref.	2013-06-05	1	0.36353	0.73733	1	1	SAMD01606006
Mj_102	Mushifugu	SJ	Sado Island, Niigata Pref.	2013-06-05	1	0.37397	0.86274	1	0	
Mj_103	Mushifugu	SJ	Sado Island, Niigata Pref.	2013-06-05	1	0.33853	0.85768	1	0	
Mj_104	Mushifugu	SJ	Sado Island, Niigata Pref.	2013-06-05	1	0.34003	0.78513	1	0	
Mj_115	Mushifugu	SJ	Sado Island, Niigata Pref.	2014-06-11	1	0.32841	0.80248	1	0	
Mj_116	Mushifugu	SJ	Sado Island, Niigata Pref.	2014-06-11	1	0.38062	0.72082	1	0	
Mj_117	Mushifugu	SJ	Sado Island, Niigata Pref.	2014-06-11	1	0.37864	0.74222	1	1	SAMD01606007
Mj_118	Mushifugu	SJ	Sado Island, Niigata Pref.	2014-06-11	1	0.33880	0.81647	1	0	
Mj_119	Mushifugu	SJ	Sado Island, Niigata Pref.	2014-06-11	1	0.37093	0.82879	1	0	
Mj_120	Mushifugu	SJ	Sado Island, Niigata Pref.	2014-06-11	1	0.35290	0.77955	1	0	
Mj_121	Mushifugu	SJ	Sado Island, Niigata Pref.	2014-06-11	1	0.36431	0.80841	0	0	
Mj_122	Mushifugu	SJ	Sado Island, Niigata Pref.	2014-06-11	1	0.39210	0.81985	0	0	
Mj_123	Mushifugu	SJ	Sado Island, Niigata Pref.	2014-06-11	1	0.36641	0.78789	0	0	
Mj_124	Mushifugu	SJ	Sado Island, Niigata Pref.	2014-06-11	1	0.36630	0.86284	0	0	
Mj_125	Mushifugu	SJ	Sado Island, Niigata Pref.	2014-06-11	1	0.36592	0.79356	0	0	
Mj_126	Mushifugu	SJ	Sado Island, Niigata Pref.	2014-06-11	1	0.36075	0.73700	0	0	
Mj_127	Mushifugu	SJ	Sado Island, Niigata Pref.	2014-06-11	1	0.36676	0.82463	0	0	
Mj_128	Mushifugu	SJ	Sado Island, Niigata Pref.	2014-06-11	1	0.35058	0.77783	0	0	
Mj_129	Mushifugu	SJ	Sado Island, Niigata Pref.	2014-06-11	1	0.32652	0.74030	0	0	
Mj_130	Mushifugu	SJ	Sado Island, Niigata Pref.	2014-06-11	1	0.34724	0.69299	0	0	
Mj_131	Mushifugu	SJ	Sado Island, Niigata Pref.	2014-06-11	1	0.37541	0.83735	0	0	
Mj_132	Mushifugu	SJ	Sado Island, Niigata Pref.	2014-06-11	1	0.34925	0.84632	0	0	
Mj_133	Mushifugu	SJ	Sado Island, Niigata Pref.	2014-06-11	1	0.34671	0.80918	0	0	
Mj_134	Mushifugu	SJ	Sado Island, Niigata Pref.	2014-06-11	1	0.34762	0.75395	0	0	
Mj_135	Mushifugu	SJ	Sado Island, Niigata Pref.	2014-06-11	1	0.36334	0.74283	0	0	
Mj_163	Mushifugu	SJ	Sado Island, Niigata Pref.	2015-06-03	1	0.34904	0.82190	1	0	
Mj_164	Mushifugu	SJ	Sado Island, Niigata Pref.	2015-06-03	1	0.39551	0.85878	1	1	SAMD01606008
Mj_165	Mushifugu	SJ	Sado Island, Niigata Pref.	2015-06-03	1	0.33854	0.73100	1	0	
Mp_001	Mushifugu	PO	Mie Pref.	NA	1	0.34591	0.85613	1	0	
Mp_007	Mushifugu	PO	Shimoda, Shizuoka Pref.	2000-06-19	0	NA	NA	1	0	
Mp_008	Mushifugu	PO	Ito, Shizuoka Pref.	2000-06-15	0	NA	NA	1	0	
Mp_009	Mushifugu	PO	Shimoda, Shizuoka Pref.	2002-09-02	0	NA	NA	1	0	
Mp_010	Mushifugu	PO	Mie Pref.	2004-05-01	0	NA	NA	1	0	
Mp_011	Mushifugu	PO	Minamiise, Mie Pref.	2004-10-08	0	NA	NA	1	0	
Mp_012	Mushifugu	PO	Minamiise, Mie Pref.	2007-02-13	1	0.38956	0.71393	1	0	
Mp_013	Mushifugu	PO	Minamiise, Mie Pref.	2007-02-13	1	0.40764	0.73519	1	1	SAMD01606009
Mp_014	Mushifugu	PO	Minamiise, Mie Pref.	2007-02-13	1	0.37515	0.77308	1	1	SAMD01606010
Mp_015	Mushifugu	PO	Minamiise, Mie Pref.	2007-02-13	1	0.38042	0.78133	1	0	
Mp_059	Mushifugu	PO	Minamiise, Mie Pref.	2007-12	1	0.38173	0.81340	0	0	
Mp_060	Mushifugu	PO	Minamiise, Mie Pref.	2007-12	1	0.33749	0.72697	0	0	
Mp_061	Mushifugu	PO	Kushimoto, Wakayama Pref.	2008-05-01	0	NA	NA	1	0	
Mp_062	Mushifugu	PO	Kushimoto, Wakayama Pref.	2008-05-01	0	NA	NA	1	1	SAMD01606011

ID	Species	Population	Location	Date	Image	Lightness	Complexity	D-loop	NGS	BioSample
Mp_063	Mushifugu	PO	Kushimoto, Wakayama Pref.	2008-06-01	0	NA	NA	1	1	SAMD01606012
Mp_064	Mushifugu	PO	Kushimoto, Wakayama Pref.	2008-06-01	0	NA	NA	1	0	
Mp_114	Mushifugu	PO	Minamiise, Mie Pref.	2014-01	1	0.39784	0.80500	1	1	SAMD01606013
Mp_178	Mushifugu	PO	Minamiise, Mie Pref.	2016-11-07	1	0.38672	0.79041	1	0	
Mp_204	Mushifugu	PO	Minamiise, Mie Pref.	2017-04-13	1	0.34706	0.75273	1	0	
Mp_205	Mushifugu	PO	Minamiise, Mie Pref.	2017-04-13	1	0.35346	0.83292	1	1	SAMD01606014
Mp_206	Mushifugu	PO	Minamiise, Mie Pref.	2017-04-13	1	0.30181	0.75870	1	0	
Mp_207	Mushifugu	PO	Minamiise, Mie Pref.	2017-04-13	1	0.35953	0.78125	1	0	
Mp_208	Mushifugu	PO	Minamiise, Mie Pref.	2017-04-13	1	0.33713	0.76746	1	1	SAMD01606015
Mp_209	Mushifugu	PO	Minamiise, Mie Pref.	2017-04-13	1	0.36613	0.77818	1	0	
Mp_210	Mushifugu	PO	Minamiise, Mie Pref.	2017-04-13	1	0.33626	0.63840	1	0	
Mp_211	Mushifugu	PO	Minamiise, Mie Pref.	2017-04-13	1	0.34798	0.84888	1	0	
Mp_212	Mushifugu	PO	Minamiise, Mie Pref.	2017-04-13	1	0.34801	0.70902	1	0	
Mp_213	Mushifugu	PO	Minamiise, Mie Pref.	2017-04-13	1	0.36483	0.80988	1	0	
Mp_214	Mushifugu	PO	Minamiise, Mie Pref.	2017-04-13	1	0.36761	0.83052	1	0	
Mp_215	Mushifugu	PO	Shima, Mie Pref.	NA	1	0.45159	0.86856	1	0	
Kj_020	Komonfugu	SJ	Shimonoseki, Yamaguchi Pref.	2006-06-18	0	NA	NA	1	1	SAMD01606016
Kj_021	Komonfugu	SJ	Shimonoseki, Yamaguchi Pref.	2006-06-18	0	NA	NA	1	0	
Kj_022	Komonfugu	SJ	Shimonoseki, Yamaguchi Pref.	2006-06-18	0	NA	NA	1	0	
Kj_023	Komonfugu	SJ	Shimonoseki, Yamaguchi Pref.	2001-11-20	0	NA	NA	1	0	
Kj_025	Komonfugu	SJ	Noto, Ishikawa Pref.	2000-09-19	0	NA	NA	1	0	
Kj_026	Komonfugu	SJ	Sado Island, Niigata Pref.	2006-11	1	0.33507	0.46441	1	1	SAMD01606017
Kj_027	Komonfugu	SJ	Sado Island, Niigata Pref.	2006-11	1	0.37585	0.48486	1	0	
Kj_066	Komonfugu	SJ	Kasumi, Hyogo Pref.	2012-05-26	1	0.41264	0.49133	1	1	SAMD01606018
Kj_067	Komonfugu	SJ	Kasumi, Hyogo Pref.	2012-06-28	1	0.30840	0.56024	1	1	SAMD01606019
Kj_068	Komonfugu	SJ	Kasumi, Hyogo Pref.	2012-06-28	1	0.33938	0.31547	1	0	
Kj_094	Komonfugu	SJ	Sado Island, Niigata Pref.	2013-06-05	1	0.26557	0.38545	1	1	SAMD01606020
Kj_095	Komonfugu	SJ	Sado Island, Niigata Pref.	2013-06-05	1	0.36737	0.53566	1	0	
Kj_105	Komonfugu	SJ	Sado Island, Niigata Pref.	2013-06-05	1	0.27596	0.37426	1	0	
Kj_137	Komonfugu	SJ	Kasumi, Hyogo Pref.	2014-11-27	1	0.35868	0.40148	1	0	
Kj_138	Komonfugu	SJ	Kasumi, Hyogo Pref.	2014-11-27	1	0.27968	0.38142	1	0	
Kj_139	Komonfugu	SJ	Kasumi, Hyogo Pref.	2014-11-27	1	0.26623	0.32911	1	1	SAMD01606021
Kj_140	Komonfugu	SJ	Kasumi, Hyogo Pref.	2014-11-27	1	0.29945	0.40922	1	0	
Kj_141	Komonfugu	SJ	Kasumi, Hyogo Pref.	2014-11-27	1	0.31324	0.46638	1	0	
Kj_166	Komonfugu	SJ	Sado Island, Niigata Pref.	2015-06-03	1	0.24164	0.33579	1	1	SAMD01606022
Kj_167	Komonfugu	SJ	Sado Island, Niigata Pref.	2015-06-03	1	0.30154	0.39637	1	0	
Kj_170	Komonfugu	SJ	Sado Island, Niigata Pref.	2016-06-08	1	0.24975	0.45372	1	0	
Kj_171	Komonfugu	SJ	Sado Island, Niigata Pref.	2016-06-08	1	0.31716	0.46263	1	0	
Kj_172	Komonfugu	SJ	Sado Island, Niigata Pref.	2016-06-08	1	0.25592	0.45525	1	0	
Kj_173	Komonfugu	SJ	Sado Island, Niigata Pref.	2016-06-08	1	0.31797	0.43912	1	0	
Kp_018	Komonfugu	PO	Minamiise, Mie Pref.	2006-05	1	0.33211	0.33792	1	0	
Kp_019	Komonfugu	PO	Minamiise, Mie Pref.	2006-05	1	0.30167	0.45874	1	1	SAMD01606023
Kp_024	Komonfugu	PO	Kosai, Shizuoka Pref.	2000-04-26	0	NA	NA	1	0	
Kp_106	Komonfugu	PO	Minamiise, Mie Pref.	2014-01	0	NA	NA	1	0	
Kp_111	Komonfugu	PO	Minamiise, Mie Pref.	2014-01	1	0.41895	0.42022	1	0	
Kp_136	Komonfugu	PO	Atsumi Peninsula, Aichi Pref.	2014-03	1	0.32450	0.30112	1	1	SAMD01606024
Kp_179	Komonfugu	PO	Kobe, Hyogo Pref.	2013-09-18	1	0.31532	0.45372	1	1	SAMD01606025
Kp_180	Komonfugu	PO	Kobe, Hyogo Pref.	2013-10	0	NA	NA	1	1	SAMD01606026
Kp_181	Komonfugu	PO	Minamiise, Mie Pref.	2014-01	0	NA	NA	1	0	
Kp_182	Komonfugu	PO	Ashiya, Hyogo Pref.	2014-11	0	NA	NA	1	1	SAMD01606027
Kp_183	Komonfugu	PO	Minamiise, Mie Pref.	2017-01-04	1	0.31593	0.35152	1	0	
Kp_184	Komonfugu	PO	Omaezaki, Shizuoka Pref.	2017-04-05	1	0.39978	0.54015	1	0	
Kp_185	Komonfugu	PO	Omaezaki, Shizuoka Pref.	2017-04-05	1	0.38323	0.47244	1	0	
Kp_186	Komonfugu	PO	Omaezaki, Shizuoka Pref.	2017-04-05	1	0.38969	0.46152	1	0	
Kp_187	Komonfugu	PO	Omaezaki, Shizuoka Pref.	2017-04-05	1	0.26907	0.41819	1	0	
Kp_188	Komonfugu	PO	Omaezaki, Shizuoka Pref.	2017-04-05	1	0.33514	0.47969	1	0	
Kp_189	Komonfugu	PO	Omaezaki, Shizuoka Pref.	2017-04-05	1	0.33051	0.48599	1	0	
Kp_190	Komonfugu	PO	Omaezaki, Shizuoka Pref.	2017-04-05	1	0.31140	0.29239	1	1	SAMD01606028
Kp_191	Komonfugu	PO	Omaezaki, Shizuoka Pref.	2017-04-05	1	0.40901	0.45122	1	0	
Kp_192	Komonfugu	PO	Omaezaki, Shizuoka Pref.	2017-04-05	1	0.35369	0.38876	1	0	
Kp_193	Komonfugu	PO	Omaezaki, Shizuoka Pref.	2017-04-05	1	0.36721	0.35492	1	0	
Kp_194	Komonfugu	PO	Omaezaki, Shizuoka Pref.	2017-04-05	1	0.37241	0.42336	1	1	SAMD01606029
Kp_195	Komonfugu	PO	Omaezaki, Shizuoka Pref.	2017-04-05	1	0.35863	0.38328	1	0	
Kp_196	Komonfugu	PO	Omaezaki, Shizuoka Pref.	2017-04-05	1	0.35372	0.41949	1	0	

ID	Species	Population	Location	Date	Image	Lightness	Complexity	D-loop	NGS	BioSample
Kp_197	Komonfugu	PO	Omaezaki, Shizuoka Pref.	2017-04-05	1	0.35868	0.45294	1	0	
Kp_198	Komonfugu	PO	Omaezaki, Shizuoka Pref.	2017-04-05	1	0.33037	0.39701	1	0	
Kp_199	Komonfugu	PO	Omaezaki, Shizuoka Pref.	2017-04-05	1	0.34869	0.48472	1	0	
Kp_200	Komonfugu	PO	Omaezaki, Shizuoka Pref.	2017-04-05	1	0.33672	0.44439	1	0	
Kp_201	Komonfugu	PO	Omaezaki, Shizuoka Pref.	2017-04-05	1	0.31477	0.41203	1	0	
Kp_202	Komonfugu	PO	Omaezaki, Shizuoka Pref.	2017-04-05	1	0.35426	0.46591	1	0	
Kp_203	Komonfugu	PO	Omaezaki, Shizuoka Pref.	2017-04-05	1	0.30777	0.47921	1	0	
Kp_221	Komonfugu	PO	Akashi, Hyogo Pref.	2022-10-20	1	0.28948	0.31914	0	0	
Kp_222	Komonfugu	PO	Akashi, Hyogo Pref.	2022-10-20	1	0.33017	0.40034	0	0	
Kp_223	Komonfugu	PO	Akashi, Hyogo Pref.	2022-10-20	1	0.32784	0.57815	0	0	
Kp_224	Komonfugu	PO	Akashi, Hyogo Pref.	2022-10-20	1	0.32590	0.42067	0	0	
Kp_225	Komonfugu	PO	Akashi, Hyogo Pref.	2022-10-20	1	0.34308	0.42376	0	0	
Kp_226	Komonfugu	PO	Akashi, Hyogo Pref.	2022-10-20	1	0.32073	0.41930	0	0	
Kp_227	Komonfugu	PO	Akashi, Hyogo Pref.	2022-10-20	1	0.33900	0.50682	0	0	
Kp_228	Komonfugu	PO	Akashi, Hyogo Pref.	2022-10-22	1	0.37175	0.44518	0	0	
Kp_229	Komonfugu	PO	Akashi, Hyogo Pref.	2022-10-22	1	0.35913	0.49465	0	0	
Kp_230	Komonfugu	PO	Akashi, Hyogo Pref.	2022-10-22	1	0.33077	0.43145	0	0	
Kp_231	Komonfugu	PO	Akashi, Hyogo Pref.	2022-10-22	1	0.23390	0.39353	0	0	
Kp_232	Komonfugu	PO	Akashi, Hyogo Pref.	2022-10-22	1	0.35656	0.41060	0	0	
Kp_233	Komonfugu	PO	Akashi, Hyogo Pref.	2022-10-22	1	0.30452	0.37941	0	0	
Kp_234	Komonfugu	PO	Akashi, Hyogo Pref.	2022-10-22	1	0.30319	0.37833	0	0	
Kp_235	Komonfugu	PO	Akashi, Hyogo Pref.	2022-10-22	1	0.38216	0.47595	0	0	
Kp_236	Komonfugu	PO	Akashi, Hyogo Pref.	2022-10-22	1	0.35586	0.48576	0	0	

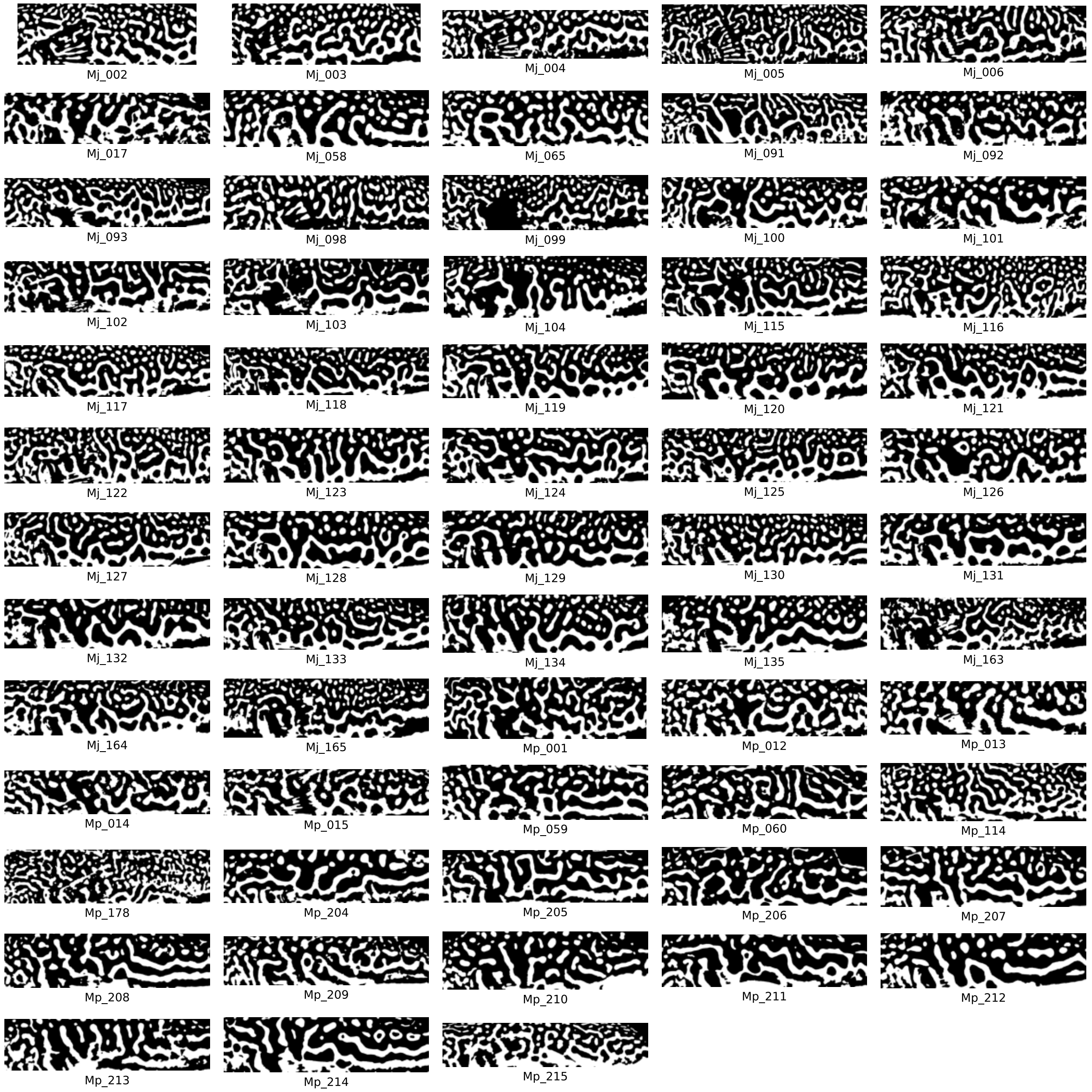

Supplementary Figure S1: Body patterns of mushifugu (*T. exascurus*).

Binarized images used for quantitateve analysis of body pattern complexity.

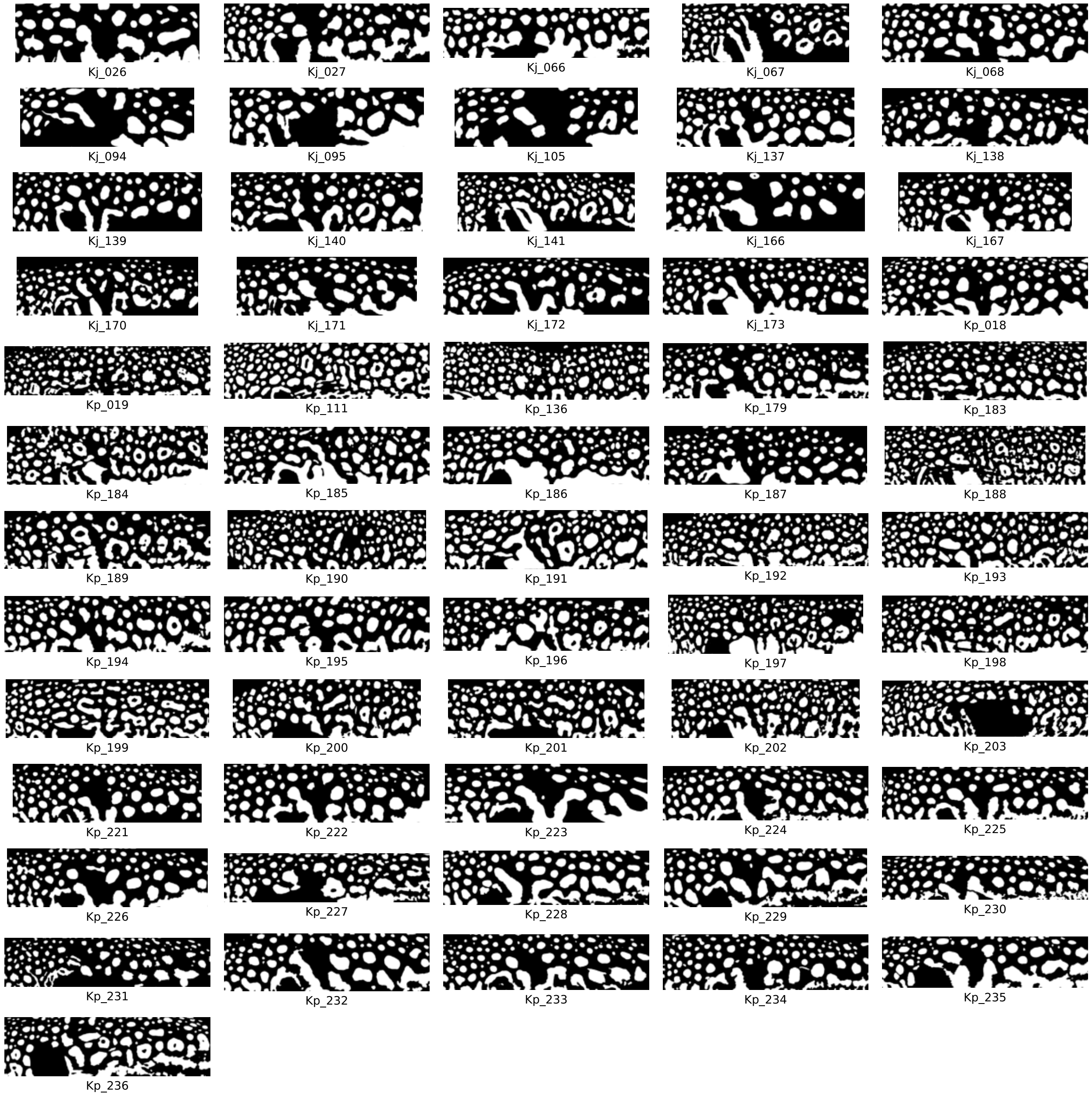

Supplementary Figure S2: Body patterns of komonfugu (*T. flavipterus*).

Binarized images used for quantitateve analysis of pattern complexity.

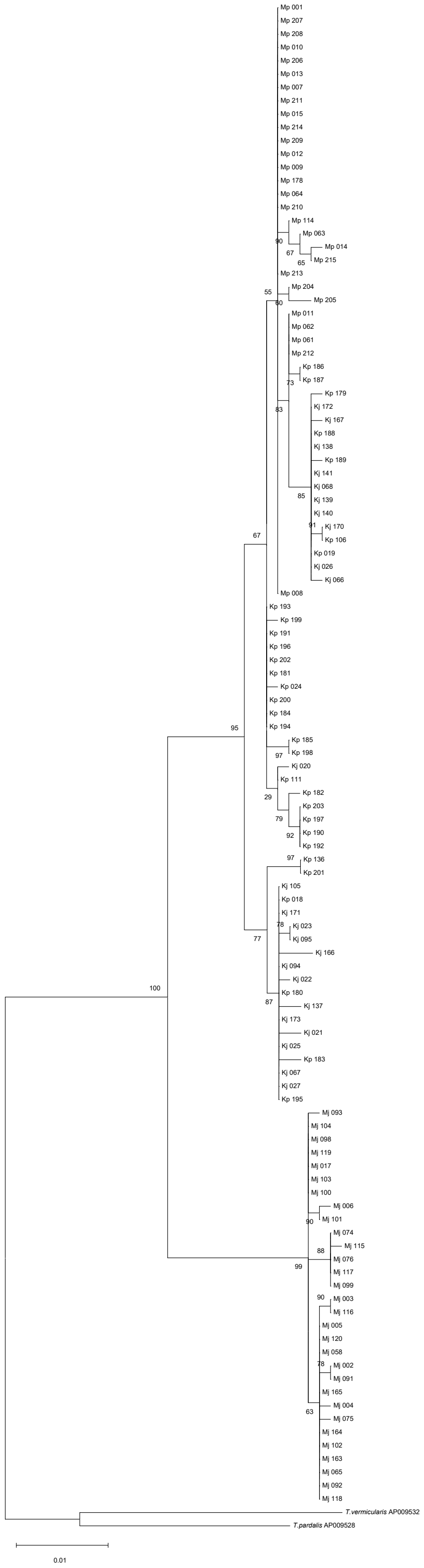

Supplementary Figure S3: Maximum-likelihood tree of mtDNA D-loop sequences. The tree was inferred in MEGA 12 (Tamura–Nei model; 500 bootstrap replicates); bootstrap support values (%) are shown on branches. The tree was rooted using *T. vermicularis* and *T. pardalis* as outgroups.

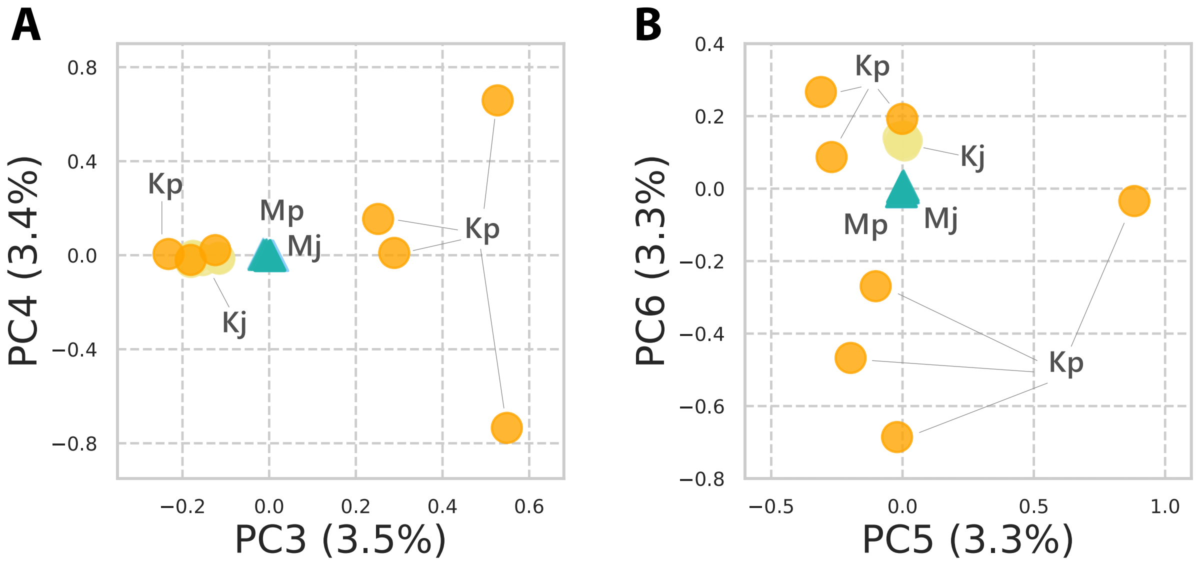

Supplementary Figure S4: Principal component analysis (PCA) of genome-wide SNP variation.

(A) Distribution of samples along the third and fourth principal components (PC3, 3.5%; PC4, 3.4%). (B) Distribution along the fifth and sixth principal components (PC5, 3.3%; PC6, 3.3%). Abbreviations: Mj, mushifugu (Sea of Japan); Mp, mushifugu (Pacific coast); Kj, komonfugu (Sea of Japan); Kp, komonfugu (Pacific coast).
